# Weight loss outcomes achieved by adults accessing an online programme offered as part of Public Health England’s ‘Better Health’ campaign

**DOI:** 10.1186/s12889-022-13847-w

**Published:** 2022-07-30

**Authors:** Josef Toon, Martina Geneva, Paul Sharpe, Jacquie Lavin, Sarah Bennett, Amanda Avery

**Affiliations:** 1 Nutrition, Health & Research Team, Slimming World, Alfreton, UK; 2 Division of Food, Nutrition & Dietetics, University of Nottingham, Nottingham, UK

**Keywords:** Weight management, online digital programme, adults, service evaluation

## Abstract

Effective use of health technology may offer a scalable solution to the obesity pandemic. Online digital programmes provide a convenient and flexible way for more people to access regular support. This service evaluation aims to determine whether adults accessing an online weight management programme via a national campaign are successful in losing weight.

Data was analysed for adults registering with Slimming World’s online programme using a discounted membership offered as part of PHE’s ‘Better Health’ campaign between July and December 2020. Last-weight carried forward was used to calculate weight outcomes for participants who had the opportunity to complete 12-weeks and recorded ≥ one weight besides baseline. Engagement was determined using number of online weekly weights recorded with high engagers having weight data for ≥ 9 occasions. Socioeconomic status was assessed using postcode data. Resubscription and uploaded weight data were used to determine numbers who continued beyond the offer period.

Twenty-seven thousand two hundred forty-eight adults (5.3% males) with mean age 41.0 ± 11.4 years met inclusion criteria. Mean baseline BMI was 33.4 ± 6.8 kg/m^2^ (29.2% 30–34.9, 18.3% 35–39.9 and 15.1% > 40 kg/m^2^). Mean weight loss at 12 weeks was 2.7 (± 3) kg representing a mean loss of 3% (± 3.1) body weight with 42.3% achieving ≥ 3% and 22.1% weight loss ≥ 5%. Median number of weigh-ins was six. Men had greater weight losses compared to women (*p* < 0.001). High engagers, both men and women, achieved greater weight losses (*p* < 0.001). Absolute weight loss was associated with joining BMI (*r*_s_ = -0.15, *p* < 0.001) but for % weight change only small differences were seen (max effect size = 0.03) with no differences in weight change for high engagers between different baseline BMI categories (*p* > 0.05). 30.9% were in the lowest two IMD quintiles and absolute and percentage weight change did not differ across deprivation quintiles (*p* > 0.05). 34.9% continued to access the online support after the offer period.

This service evaluation shows that an online programme, offered as part of a national campaign, can offer effective support to a large number of people with different starting BMIs and from different socioeconomic backgrounds. An increased level of engagement leads to better weight losses.

## Introduction

The effective use of health technology has become particularly important during the COVID pandemic where the provision of in-person group support has been challenging due to restrictions placed upon in-person support groups. In terms of weight management, there is a further growing interest in the role of online weight management support as a scalable solution to the obesity pandemic, offering a convenient and flexible way for people seeking to manage their weight to access regular support thus providing greater reach than traditional services. Ideally, online weight management platforms need to be able to support people of different ages, genders, ethnicities and socioeconomic backgrounds and to be able to replicate the peer support which can be achieved through in-person groups.

Community weight management programmes such as offered by Slimming World provide online weight management support for most adults, building upon the support that has been provided over fifty years within their in-person groups. The support provided from Slimming World’s online programme includes access to an online community and live member events, with structured behaviour change support from the start of a new member’s weight loss journey. Members are encouraged to log their self-reported weight on a weekly basis and set personalised goals with weekly support tailored according to the members weekly weight changes and level of confidence in their ability to make lifestyle changes. Members have access to webinars, recipes, meal plans, features and various tools including food and activity planners and diaries to self-monitor their lifestyle changes. Behavioural change strategies help to motivate, prevent relapse and awards are available to encourage weight loss success (https://www.slimmingworld.co.uk/what-happens-online). Members have access to an online support team and there is registered nutrition and dietetic advice for people who need additional expert guidance.

The online support provided by Slimming World has been included as part of a national public health campaign by Public Health England. Better Health aims to help the nation get healthier by providing a selection of resources and tools which offer advice and support on various topics such as losing weight, getting active, quitting smoking, reducing alcohol intake and having good mental well-being. The national campaign provides members of the public with discounted membership of weight management programmes such as Slimming World (https://www.nhs.uk/better-health/lose-weight/). Adults signing up to Slimming World through the Better Health campaign promotion are able to choose three months of online support and then continue self-funding either online or in-person group support for as long as they wish to.

The National Institute for Health & Clinical Excellence, NICE (2014) [1], recommend that weight management programmes are available for a minimum of three months and use best practice guidance which suggests successful programmes are likely to lead to an average weight loss of at least 3%, with at least 30% of participants losing at least 5% of their initial weight after three months of the intervention.

There is currently limited data available to determine the effectiveness of online weight management support and no data evaluating the effectiveness when offered via a national public health campaign.

Hence the aim of this service evaluation is to determine whether adults accessing online support via a national campaign are successful in losing weight with a 3% weight loss considered to be successful [1].

## Methods

Data was analysed for adults, (18 -80yrs), who signed up for Slimming World’s online programme using a unique discount code offered as part of Public Health England’s ‘Better Health’ campaign between July and December 2020. The unique code offered discount (25–33%) off a 3-calendar month membership to the online programme for adults with a self-reported baseline body mass index (BMI) of ≥ 23 kg/m^2^. This lowest acceptable BMI was agreed with PHE to include people from certain ethnic backgrounds who may benefit from weight management despite having a BMI that would otherwise be considered as being in the healthy range. A last-weight observed carried forward model was used to calculate BMI and weight change outcomes for those adults with a recorded joining date and who had the opportunity to complete 12-weeks at the time of data extraction (January 2021). The inclusion criteria also required the provision of self-reported age and height data, plus weight on at least one occasion besides their joining (baseline) data. People with a BMI > 90 kg/m^2^ and outlying weight change data, defined as losing > 5% of their baseline weight within one week (apart from week 1), were excluded.

Level of engagement was determined by the number of weekly weights recorded on the online platform with high engagers defined as having provided weight data for nine or more occasions within their first 12 weeks/3 months of the offer (only one weight per week can be reported on the online platform). Socioeconomic status was assessed using postcode data to calculate the Index of Multiple Deprivation. Resubscription, alongside further weight data being uploaded up to four weeks outside the initial subscription period, were used to determine the numbers who continued to access the online support beyond the offer period.

Data were described using descriptive statistics and a multiple logistic regression model was fitted to predict the likelihood of people achieving a 3% weight loss.

Evaluation was an integral component of the partnership between PHE and the individual Better Health service providers, with all providers expected to provide data to monitor the service being offered. As a service evaluation, no ethical approval was requested. All adults signing up to Slimming World membership consent for their data recorded to be used for evaluation and service improvement purposes.

## Results

**Fig. 1:**
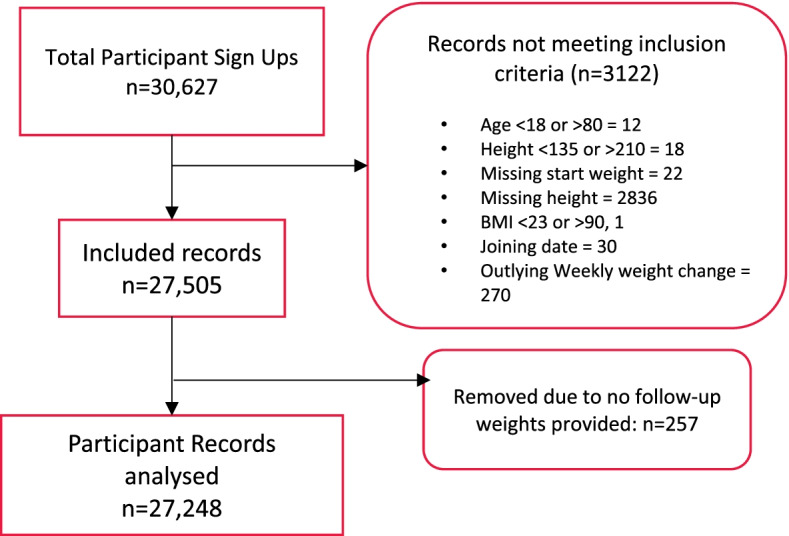
CONSORT flow diagram for online members joining via the Better Health programme

Twenty-seven thousand two hundred forty-eight adults (5.3% males) residing in the UK and Ireland met the study inclusion criteria and had the opportunity to access 12 weeks of online support after accessing the Better Health campaign discounted membership (Fig. 1). Many who did not report a height also did not provide a follow-up weight. Only 0.93% (257/27,505) were removed from the data analysis as they had no follow-up weight reported.

For those adults meeting the inclusion criteria, the mean age was 41.0 ± 11.4 years. The majority (87.0%) were below the age of 55 years. Mean BMI at baseline was 33.4 ± 6.8 kg/m^2^ with 29.2% having a BMI in the 30–34.9 kg/m^2^ category, 18.3% in the 35–39.9 kg/m^2^ and 15.1% in the > 40 kg/m^2^ category.

The mean absolute weight loss at 12 weeks was 2.7 (± 3.0) kg representing a mean loss of 3% (± 3.1) body weight with 42.3% achieving a weight loss of ≥ 3% and 22.1% achieving a weight loss of ≥ 5%. 88.9% of participants achieved a reported weight loss by the end of the 12-week period. The median number of weigh-ins was six.

### Influence of gender and level of engagement

Men had greater weight loss in all measures of weight change compared to women (-4.2 ± 4.1 v. -2.6 ± 2.9 kg; *p* < 0.001) (Table 1). High engagers, defined as those recording ≥ 9 follow-up weights, achieved greater weight losses and even among high engagers, there was a significant difference in weight loss between the genders (-6.3 ± 4.6 v. -4.1 ± 3.3 kg; *p* < 0.001).

**Table 1:** Weight loss outcomes according to gender and level of engagement

	All study population				High Engagers			
	Total (*n* = 27,248)	Male (*n* = 1453)	Female (*n* = 25,795)	Sig	Total (*n* = 9531)	Male (*n* = 551)	Female (*n* = 8980)	Sig
Change in BMI	-1.0 ± 1.1	-1.3 ± 1.3	-1 ± 1.1	***	-1.5 ± 1.2	-2.0 ± 1.4	-1.5 ± 1.2	***
Change in Kg	-2.7 ± 3	-4.2 ± 4.1	-2.6 ± 2.9	***	-4.2 ± 3.4	-6.3 ± 4.6	-4.1 ± 3.3	***
Change in %weight	-3.0 ± 3.1	-3.8 ± 3.5	-2.9 ± 3.1	***	-4.7 ± 3.5	-5.6 ± 3.8	-4.6 ± 3.5	***

^***^ = *p* < 0.001, ** = *p* < 0.01, * = *p* < 0.05

### Influence of baseline BMI category

Weight loss data by joining BMI categories can be seen in Table 2. Correlational analysis showed absolute weight loss was associated with joining BMI, with greater weight loss in people with higher baseline BMI values (*r*_s_ = -0.15, *p* < 0.001). A Kruskal–Wallis test of percentage weight change against joining BMI category was significant, although post-hoc Wilcoxon tests showed the only significant differences were in contrasts to 35- < 40 BMI group and the magnitude of these differences were very small (max effect size = 0.03). There were no significant differences in either absolute or percentage weight change for high engagers between the different baseline BMI categories.

**Table 2:** Weight outcomes by joining BMI category for all members and high engagers (defined as those recording ≥ 9 follow-up weights)

	**BMI Category**	**Change in BMI**	**Change in Kg**	**Change in %weight**
**All study population**	23–25 (*n* = 1764)	-0.7 ± 0.7	-2.1 ± 2.1	-3.1 ± 3.1
	25- < 30 (*n* = 8426)	-0.8 ± 0.9	-2.3 ± 2.5	-3 ± 3.2
	30- < 35 (*n* = 7963)	-1 ± 1	-2.6 ± 2.8	-2.9 ± 3.2
	35- < 40 (*n* = 4987)	-1.1 ± 1.2	-2.9 ± 3.2	-2.9 ± 3.1
	40 + (*n* = 4108)	-1.3 ± 1.4	-3.6 ± 3.8	-2.9 ± 3
**High engagers**	23–25 (*n* = 619)	-1.1 ± 0.9	-3.1 ± 2.4	-4.6 ± 3.5
	25- < 30 (*n* = 3113)	-1.3 ± 1	-3.5 ± 2.8	-4.6 ± 3.5
	30- < 35 (*n* = 2770)	-1.5 ± 1.2	-4.2 ± 3.3	-4.7 ± 3.6
	35- < 40 (*n* = 1680)	-1.7 ± 1.3	-4.8 ± 3.6	-4.7 ± 3.5
	40 + (*n* = 1349)	-2.1 ± 1.6	-5.9 ± 4.4	-4.7 ± 3.5

### Levels of deprivation

Using available postcode data (*n* = 22,288, 81.7% of dataset), the percentage of people accessing the online support are presented according to quintile of deprivation where quintile 1 represents the highest level of deprivation. 30.9% were in the lowest two quintiles.

A Kruskal–Wallis test found that absolute and percentage weight change did not differ significantly across deprivation quintiles (*p* > 0.05) (Table 3).

**Table 3:** Mean weight loss outcomes by deprivation quintile

IMD Quintile	Change in BMI	Change in Kg	Change in % weight
1 (*n* = 2934) / (13.2%)	-1 ± 1.1	-2.7 ± 3	-2.8 ± 3
2 (*n* = 3951) / (17.7%)	-1 ± 1.1	-2.7 ± 3	-2.9 ± 3.1
3 (*n* = 4851) / (21.8%)	-1 ± 1.1	-2.7 ± 2.9	-2.9 ± 3.1
4 (*n* = 5326) / (23.9%)	-1 ± 1.1	-2.7 ± 2.9	-3 ± 3.1
5 (*n* = 5226) / (23.4%)	-1 ± 1.1	-2.7 ± 3	-3.1 ± 3.2

### Predicting a 3% weight loss

A multiple logistic regression model was fitted to predict the likelihood of achieving a 3% weight loss. The model was trained on 80% of the data and used age, joining BMI, gender, number of times self-reported weight was recorded and deprivation quintile as predictors. The initial model did not find deprivation quintile or joining BMI to be significant predictors of achieving a 3% weight loss and were dropped from the model. The updated model showed that the odds of achieving a 3% weight loss were 1.54 times higher for men versus women and 1.28 times greater for each additional weight reported. The odd ratios and confidence intervals of the model are shown in Table 4.

**Table 4:** Odd Ratios and Confidence Interval of achieving a 3% weight loss

	Odd Ratios	Lower CI	Upper CI
Intercept	0.181	0.159	0.206
Age	0.992	0.990	0.995
Gender (Male)	1.538	1.332	1.776
No. weights recorded at 12 weeks	1.276	1.264	1.288

$$\mathrm{log}-\mathrm{odds }= -1.54 - 0.007(\mathrm{age}) + 0.439(\mathrm{Male}) + 0.243(\mathrm{No}.\mathrm{ of weights recorded})$$

The model was tested against the remaining test data and was found to have a 68.5% accuracy rate. The area under the ROC was 0.73.

### Continued access to the online programme

The investigation as to how many of the sample may have continued to access the online support beyond the initial 3 month offer period suggests that 34.9% continued (as determined by weight outcomes being uploaded up to four weeks after the PHE offer time period). The gender profile was very similar to the initial group who engaged with 5.2% men continuing to access the support v. 5.3%. The mean age of people continuing to access support was 42.1 ± 11.3 years (v. 41.0 ± 11.4) and they had a mean BMI of 33.2 ± 6.8 kg/m^2^ (v. 33.4 ± 6.8) at the start of the evaluation period. Those continuing achieved a BMI change of -1.4 ± 1.2 and a mean weight loss of 4.2 ± 3.6% (v.3.0 ± 3.1).

## Discussion

This service evaluation reports the efficacy of a 12-week weight management programme offered online, incorporating some of the behavioural change support that would normally be offered within in-person groups. The service evaluation is bespoke to those adults who accessed the online support via Public Health England’s Better Health national campaign where they were able to obtain a discounted rate for their membership.

Over 25,000 UK adults accessed Slimming World’s online support during the three-month study period with 99% providing more than one weight change emphasising the representativeness of the data reported. Over 60% of the study population were people living with obesity and more than 20% achieved a weight loss of 5% or greater. The mean weight loss of 3% over this initial 12-week membership meets the recommendations for weight management programmes advised by the NICE guidelines (NICE, 2014) [1]. The mean absolute weight loss reported is 1 kg greater than the weight loss reported in the OWLET trial [2] where participants also accessed online weight management support although the study population in our evaluation had selected to access the online support rather than being randomised to the support and referred via primary care. A meta-analysis of 61 studies found similar results with greater weight loss (kg) post intervention in digital weight loss interventions compared to minimal or standard care (MD − 2.70 [− 3.33, − 2.08], *p* < 0.001) [3].

Whilst men only represented just over 5% of the study population, their weight losses were greater than females. This data is in line with other studies where weight loss in males has been reported to be consistently higher than in females attending mixed-sex programmes [4, 5]. Men are also under-represented in weight loss research with a systematic review of 244 randomised controlled trials of weight management programmes reporting that only 27% of the > 95,000 participants were men [6]. The problem remains how to encourage more men to engage with weight management programmes whether online or in-person groups. Qualitative data suggests that men do not think that it is a male thing to be worrying about their weight and there is a certain amount of stigma for men unless they have a medical diagnosis or health threat and are referred to lose weight by a medical practitioner [7].

Also consistent with recent reviews [3, 8, 9], those people who were perceived as engaging more with the online support, were more successful in their level of reported weight loss. Level of engagement may be associated with greater commitment [11] and self-monitoring weight via uploading personal weight on a weekly basis could be motivating [11]. Other components of the online programme such as setting personalised goals, using the planners, food diaries, webinars, recipes and online features may have all helped to positively influence weight management and prevent relapse in the majority of participants in this study. Some people, and particularly those with higher weights at baseline, may need longer than a 12-week period to be able to feel confident that any lifestyle changes that they have made can be sustained. The online support offered by Slimming World is available for as long as people feel that they need it, with data from this study suggesting that just over a third of the sample felt that they needed the support for longer. This additional support does come at an additional financial cost to the individual and further analysis would be helpful to understand the socioeconomic profile of this group besides the subsequent weight changes achieved. Other people who engaged in the online support subsidised as part of the Better Health partnership may have gone on to have attended in-person groups offered by Slimming World but this data is not captured in the current study.

Baseline BMI did influence absolute but not percentage weight loss for both the total study population and the high engagers. Whilst important that those people joining with higher baseline BMIs were able to lose more absolute weight, a 3% weight loss may not be clinically significant where baseline BMI is over 35 kg/m^2^ and either a greater level or length of engagement needs to be encouraged to achieve a weight loss nearer 5%. It can be more difficult for people from lower socioeconomic backgrounds to achieve healthier lifestyles because of the direct and tangible costs of following a healthier diet [12] and increasing activity through the use of structured exercise programmes [13]. However female adults from the lowest socioeconomic groups present with higher levels of obesity [14] and thus may benefit more from weight management support. In terms of reducing health inequalities, it is very important that publicly funded programmes provide equity of access and if possible try and target people from more vulnerable populations including people from lower socioeconomic groups. Thus it is pleasing that almost a third of those people accessing the support were from the two most deprived groups when the IMD data was split into quintiles of deprivation. Even more important was the finding that the people who engaged from the two most deprived groups were equally likely to achieve the same levels of weight loss. This would suggest that the guidance provided through the online support was appropriately ‘pitched’ to all those engaging irrespective of their socioeconomic background.

Previous studies that have investigated the effectiveness of behavioural weight loss programmes delivered in everyday contexts suggest that commercial interventions delivered in the community are effective for achieving weight loss [15] and represent good value for money [16]. For some individuals online weight management support may lead to greater weight loss outcomes compared to in-person group support particularly in the short-term [17]. It may also be more cost effective to deliver at scale. The Slimming World online support has been designed to provide similar personal behaviour change guidance and support tools as offered in the in-person groups, including access to an online member community and live events to provide a level of peer support but some people may struggle to engage as fully as they would in person. The current data was collected during the COVID pandemic which was known to generally affected people’s eating habits and mental well-being, with a number of studies reporting that people found it more difficult to manage their weight during this time [18–20]. The jury is out as to whether greater weight loss is achieved if people have to pay for weight management services that they can afford or whether there are benefits of people being referred into a subsidised programme at no cost to themselves [21]. People accessing the Better Health programme were effectively self-funding a subsidised programme offered via PHE. Also, they had selected the Slimming World programme rather than other choices so theoretically should have been prepared to make changes.

One of the limitations of this current evaluation of the online support offered via Slimming World is that the study population was limited to those who had had time to complete the 12-week programme at the time data was downloaded. Hence the data reported is not for all adults who have accessed the Slimming World online support via Better Health. The data was downloaded at the end of the 12-week intervention and longer term follow up data would provide additional information as to whether the weight losses achieved could be further enhanced or, at the minimum, be maintained. The findings from the in-person group population are that weight losses achieved can be maintained at one year follow-up [22] and also that around half of people who were referred continue to attend after the initial 12-week referral [23].

Baseline data collection was limited to age, gender, BMI and IMD index. We did not collect data on other health parameters, use of medication, dietary and physical activity changes made or collected any qualitative data. Other studies undertaken by the Slimming World research team have found that people accessing the support make behavioural changes including positive dietary changes and increase their physical activity levels [24].

Pragmatic and scalable national solutions are required for community weight management services that can be easily accessed by people living with obesity from different socioeconomic groups. This service evaluation shows that an online programme, offered as part of a national campaign led by PHE, can provide effective support to a large number of people with different starting BMIs and from different socio-economic backgrounds. As always, an increased level of engagement can lead to better weight loss outcomes.

## Data Availability

The datasets generated and/or analysed during the current study are not publicly available due to the nature of the partnership between SW and PHE but are available from the corresponding author on reasonable request.
